# High BMI Is Associated with Changes in Peritumor Breast Adipose Tissue That Increase the Invasive Activity of Triple-Negative Breast Cancer Cells

**DOI:** 10.3390/ijms251910592

**Published:** 2024-10-01

**Authors:** Cora E. Miracle, Chelsea L. McCallister, Krista L. Denning, Rebecca Russell, Jennifer Allen, Logan Lawrence, Mary Legenza, Diane Krutzler-Berry, Travis B. Salisbury

**Affiliations:** 1 Department of Biomedical Sciences, Joan C. Edwards School of Medicine, Marshall University, Huntington, WV 25755, USA; miracle13@marshall.edu (C.E.M.); thompsonch@marshall.edu (C.L.M.); 2 Cabell Huntington Hospital Laboratory, Department of Pathology, Joan C. Edwards School of Medicine, Marshall University, Huntington, WV 25701, USA; haught5@marshall.edu (K.L.D.); rebecca.russell@chhi.org (R.R.); jennifer.allen@chhi.org (J.A.); lawrence54@marshall.edu (L.L.); 3 Edwards Comprehensive Cancer Center, Department of Oncology, Joan C. Edwards School of Medicine, Marshall University, Huntington, WV 25701, USA; legenza@marshall.edu (M.L.); krutzler@marshall.edu (D.K.-B.)

**Keywords:** triple negative breast cancer, obesity, breast cancer invasion, JAG1

## Abstract

Breast cancer is the most common cancer in women with multiple risk factors including smoking, genetics, environmental factors, and obesity. Smoking and obesity are the top two risk factors for the development of breast cancer. The effect of obesity on adipose tissue mediates the pathogenesis of breast cancer in the context of obesity. Triple-negative breast cancer (TNBC) is a breast cancer subtype within which the cells lack estrogen, progesterone, and HER2 receptors. TNBC is the deadliest breast cancer subtype. The 5-year survival rates for patients with TNBC are 8–16% lower than the 5-year survival rates for patients with estrogen-receptor-positive breast tumors. In addition, TNBC patients have early relapse rates (3–5 years after diagnosis). Obesity is associated with an increased risk for TNBC, larger TNBC tumors, and increased breast cancer metastasis compared with lean women. Thus, novel therapeutic approaches are warranted to treat TNBC in the context of obesity. In this paper, we show that peritumor breast adipose-derived secretome (ADS) from patients with a high (>30) BMI is a stronger inducer of TNBC cell invasiveness and JAG1 expression than peritumor breast ADS from patients with low (<30) BMI. These findings indicate that patient BMI-associated changes in peritumor AT induce changes in peritumor ADS, which in turn acts on TNBC cells to stimulate JAG1 expression and cancer cell invasiveness.

## 1. Introduction

Breast cancer is the most common cancer in women worldwide, with incidence rates expected to increase by 40% by the year 2040 [1]. Breast cancer is divided into four molecular subtypes: luminal A, luminal B, HER2+, and triple-negative breast cancer (TNBC) [2,3,4]. TNBC is the deadliest breast cancer subtype as it does not possess the estrogen receptor (ER+), progesterone receptor (PR+), or HER2 and is therefore resistant to targeted therapy [5]. The absence of these markers hinders the ability for clinical intervention. In addition, TNBC is known to have a high heterogeneity, making it even more difficult to treat [6]. While TNBC largely remains understudied compared to ER/PR+ breast tumors, it still accounts for 10–20% of breast cancer diagnoses [6]. TNBC tends to occur in premenopausal women under 40 years old and disproportionately occurs in women of color [7,8]. Obese black women are diagnosed with breast cancer more frequently than obese white women, and the risk for early onset TNBC is higher in obese black women than obese white women [9]. The survival after metastasis of TNBC is only 13.3 months [10]. The factors that predispose these women to such a deadly illness remain an active area of study. Recently, clinical studies have shown a link between TNBC and obesity [11,12]. One study showed that women who had been diagnosed with TNBC were more likely to be overweight/obese (odds ratio = 1.89) [13]. Further, a clinical study showed that of 183 TNBC patients, 63.7% were obese [14]. These clinical studies show a link between obesity and TNBC. However, the mechanisms by which obesity promotes the progression and incidence of TNBC are unclear.

Obesity affects one in every three adults in the United States, with the prevalence rising each year [15,16,17]. While obesity is commonly linked with diabetes and metabolic disease, obesity has also been linked to thirteen different cancers, including breast cancer [18]. Patients who are obese at the time of diagnosis have been shown to have increased breast tumor grade, larger tumor size, poor prognosis, and increased risk of metastasis [19,20,21,22]. The mechanism linking obesity to breast cancer progression is hypothesized to arise from obesity-induced pathological changes within adipose tissue (AT) that in turn alter the levels of factors that are released from AT [23,24,25,26,27]. The factors secreted from AT are collectively termed the adipose-derived secretome (ADS). The ADS consists of at least 100 proteins, cytokines, growth factors, free fatty acids, and extracellular vesicles [23,24,25]. Obesity modulates the levels of adipokines and extracellular vesicles in ADS [23,24,25]. For instance, obesity is associated with increased secretion of interleukin 6 (IL-6) and leptin and reduced secretion of adiponectin from AT [23,24,25]. Obesity-associated changes in adipokine levels in turn act on breast cancer cells to amplify signaling that promotes cancer progression [23,24,25,28]. The TNBC-promoting effect of obesity can be reversed with caloric restriction and weight loss in a murine model of TNBC with obesity [29]. Breast adipocytes and adipocyte-derived mesenchymal stem/stromal cells that are in direct contact with TNBC cells are part of the tumor microenvironment. TNBC cells secrete factors that act on nearby breast adipocytes and stromal cells to induce signaling that promotes fibroblastic properties and increases the release of inflammatory cytokines that, in turn, act on TNBC cells to promote cancer aggressiveness [30,31]. Collectively, crosstalk between adipocytes and cancer cells in the tumor microenvironment promotes cancer through mechanisms that could be promoted by obesity.

Multiple signaling pathways have been identified as poor prognostic markers of TNBC progression [32,33,34,35,36,37]. The pathways include but are not limited to phosphatidylinositol 3-kinase/mechanistic target of rapamycin (PI3K/mTOR), extracellular signal-regulated kinase (ERK), nuclear factor kappa-light-chain-enhancer of activated B cells (NFKB), and JAG1-NOTCH signaling [32,33,34,35,36,37]. ERK signaling promotes the proliferation and survival of TNBC cells [34]. JAG1-NOTCH signaling promotes TNBC cell invasiveness and stemness [33,36,37]. PI3K/mTOR signaling reduces the responsiveness of TNBC to chemotherapy [35]. NFKB signaling promotes the proliferation and invasiveness of TNBC cells [38,39]. Despite the well-established roles of these signaling pathways in TNBC, the specific roles of these pathways in TNBC in obesity require further study.

Based on these studies, we hypothesized that obesity-associated peritumor breast ADS acts on TNBC cells to induce signaling that promotes the migration and invasiveness of TNBC cells. Recently, we published an association between obesity, peritumor breast ADS, and the increased migration and invasiveness of luminal estrogen-receptor-positive breast cancer cells [28]. In this study, we extend those findings by showing that the peritumor breast ADS from patients with BMIs > 30 is a stronger promotor of TNBC cell invasiveness and JAG1 expression than peritumor breast ADS from patients with BMIs < 30. These changes were associated with significantly increased levels of leptin and IL-6 in the peritumor ADS from patients with BMIs > 30 compared with BMIs < 30. The increase in TNBC cell invasiveness in response to BMI was correlated with an increase in the expression of JAG1 in TNBC cells. JAG1, via its receptor, NOTCH1, promotes the migration and invasiveness of TNBC cells [37,40]. These new findings indicate that patient BMI-induced changes in peritumor AT promote the invasiveness and JAG1 signaling in TNBC cells.

## 2. Results

### 2.1. High (>30)-BMI-Associated Peritumor ADS Increases the Invasiveness of TNBC Cells

**Figure 1:**
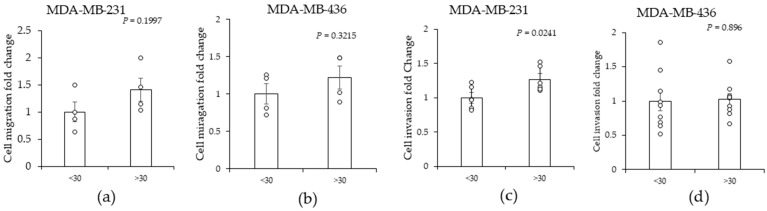
The effect of the peritumor AT-derived secretomes from individuals with different BMIs on the migration of (**a**) MDA-MB-231 and (**b**) MDA-MB-436 cells and invasion of (**c**) MDA-MB-231 and (**d**) MDA-MB-436 cells. Peritumor AT was obtained from breast cancer patients. Pieces of peritumor AT were cultured in serum-free cell culture media for 24 h. The media covering the peritumor AT was then collected as the peritumor AT-derived secretome (ADS). Peritumor ADS was diluted 1:10 in media with 0.1% FBS and applied to breast cancer cells for 24 h to assess cell migration in panels (**a**,**b**) and used as the chemoattractant for 24 h to assess cancer cell invasiveness in panels (**c**,**d**). Significant increase by BMI >30 compared with BMI <30, based on Student’s *t*-test analysis, is indicated by *p* < 0.05 (*n* = 4–9). The data are shown as the mean ± SEM (error bars).

To determine if obesity, as measured by BMI, influences the peritumor breast adipose secretome (ADS), we obtained freshly isolated breast peritumor AT samples (~100 mg) from women with breast cancer who had different BMIs. The distance from the breast AT sample to the tumor ranged from 5 to 15 cm; therefore, the AT samples were not infiltrated by cancer cells and thus not directly cancer associated but instead located near the tumor; therefore, the samples are referred to as being peritumor AT. Paraffin sections were prepared and examined by a pathologist to determine that breast adipose tissue samples were cancer free (Supplementary Figure S1). The freshly isolated AT samples were carefully cut into five equally sized pieces and cultured in serum-free cell culture media for 24 h. The peritumor breast-AT-conditioned medium, which we will refer to as AT-derived secretome (ADS), was diluted 1:10 in serum-free medium and applied to MDA-MB-231 and MDA-MB-436 TNBC cell lines, and the effect on cancer cell migration and invasiveness was assessed. To evaluate migration, we applied peritumor breast ADS to TNBC cells and quantified the migration of the TNBC cells across a “scratch” in a cell monolayer (wound healing assay). The results showed that the effect of BMI was variable; however, a trend of increased migration in response to BMI > 30 was noted as compared with BMI < 30 (Figure 1a,b). Photographs showing cells in the migration assay are shown in Supplementary Figure S2. The variability was anticipated given that the peritumor breast ADS was derived from different patients, presenting multiple donor-specific variables. Next, we assessed the effect of BMI on TNBC cell invasiveness. The invasion of MDA-MB-231 and MDA-MB-436 TNBC cells through a basement membrane towards peritumor breast ADS was assessed using a modified Boyden chamber assay. TNBC cells were plated in the upper chamber and their ability to invade through a basement membrane to a lower chamber containing peritumor breast ADS was determined. The results showed that BMIs > 30 significantly increased the invasion of TNBC cells relative to BMIs < 30 (Figure 1c). The BMI effect on TNBC invasion was cell line dependent, with MDA-MB-231 cells being the more responsive cell line compared with MDA-MB-436 (Figure 1c,d). We assessed the effect of tumor grade, and the results show that tumor grade was not significantly associated with ADS regulation of MDA-MB-231 and MDA-MB-436 invasiveness and that tumor grade was not a factor in the cell migration study (Supplementary Figure S3). These findings indicate that patient BMI is associated with changes in peritumor breast ADS, which in turn acts on TNBC cancer cells via a specific mechanism to promote cell migration and invasiveness.

### 2.2. A High BMI (≥30) Is Associated with Significant Increases in Leptin and Interleukin-6 (IL-6) in Peritumor Breast ADS

**Figure 2:**
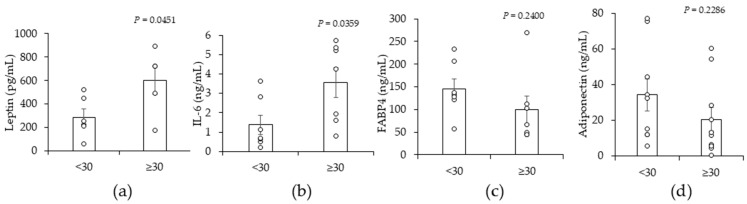
The effect of BMI on levels of leptin, interleukin-6 (IL-6), fatty acid binding protein 4 (FABP4), and adiponectin in peritumor AT-derived secretome (ADS). Peritumor AT (~100 mg) was cut into 5 small pieces and cultured in serum-free media for 24 hr. Media covering peritumor AT was collected as the peritumor ADS. The concentration of (**a**) leptin, (**b**) IL-6, (**c**) FABP4, and (**d**) adiponectin in peritumor ADS was measured by ELISA. A significant increase by BMI ≥ 30 is indicated by *p* < 0.05 (*n* = 4–7).

Next, we asked whether BMI influenced the secretion of adipokines from peritumor breast AT by assessing the concentrations of leptin, interleukin-6 (IL-6), fatty acid binding protein 4 (FABP4), and adiponectin in peritumor breast ADS. The results show peritumor breast AT explants release fatty acid-binding protein 4 (FABP4), leptin, adiponectin, and interleukin 6 (IL-6) (Figure 2a–d). The concentration of leptin and IL-6 was significantly higher in ADS from peritumor breast AT from patients with BMIs ≥ 30 compared with patients with BMIs < 30 (Figure 2a,b). BMI did not significantly alter the concentration of FABP4 or adiponectin in peritumor ADS (Figure 2c,d). We questioned whether tumor grade influenced the release of adipokines from peritumor AT. The results show that tumor grade was not significantly associated with differences in the concentrations of leptin, IL-6, FABP4, and adiponectin in peritumor ADS (Supplementary Figure S4). The BMI effect on IL-6 and leptin is consistent with the BMI effect on TNBC invasiveness (Figure 1), given that these two adipokines can stimulate TNBC cancer cell migration and invasiveness [41,42].

### 2.3. High (>30)-BMI-Associated Peritumor ADS Stimulates Increases in JAG1 in TNBC Cells

**Figure 3:**
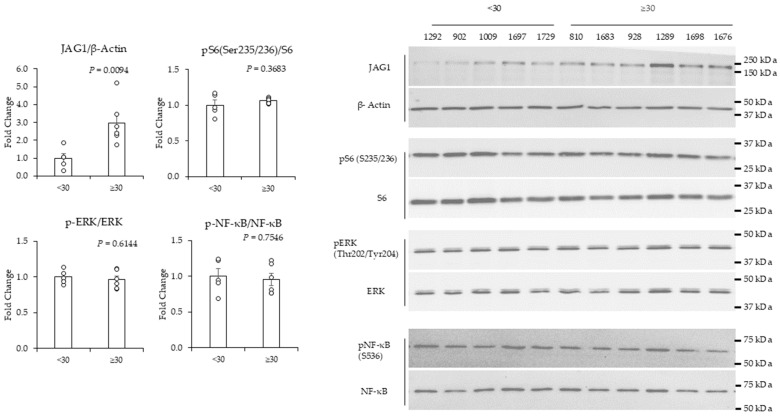
Western blot analysis of MDA-MB-231 cells treated with peritumor AT-derived secretome (ADS). Peritumor AT was obtained from 11 patients with breast cancer. Peritumor AT (~100 mg) was cut into 5 equal pieces and cultured in serum-free media for 24 hr. The media covering peritumor AT was then collected as the peritumor ADS. Peritumor ADS was diluted 1:10 in media with 0.1% FBS and applied to MDA-MB-231 cells for 24 h. Total cell lysates were analyzed by Western blotting. The expression of JAG1 protein was significantly increased in MDA-MB-231 cells treated with peritumor ADS from patients with BMIs ≥ 30 compared to peritumor-ADS from patients with BMIs < 30. JAG1 and ꞵ-actin were probed sequentially on the same blot and ꞵ-actin was used to normalize samples. Western blot analysis of the remaining proteins showed their expression was not associated with BMI. The BMIs associated with the specific patient IDs shown above the Western blot image are indicated in the Supplementary Materials (Supplementary Table S1). Results were analyzed by the student’s *t* test for statistically significant differences (*p* < 0.05). Data represent the mean signal ± SEM (error bars) (*n* = 5–6).

**Figure 4:**
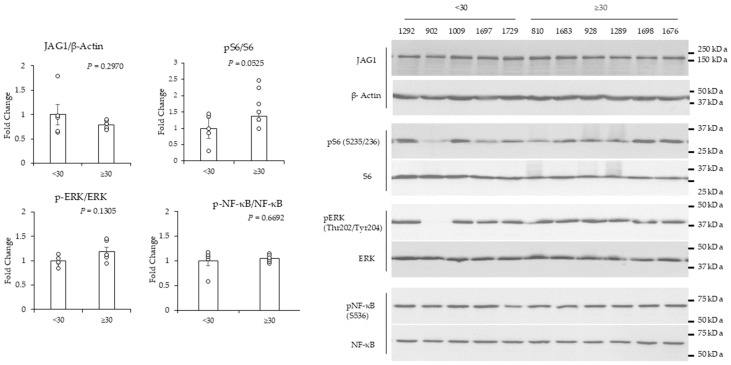
Western blot analysis of MDA-MB-436 cells treated with peritumor AT-derived secretome (ADS). Peritumor AT was obtained from 11 patients with breast cancer. Pieces of peritumor AT (~100 mg) were cultured in cell culture media for 24 hr. The media covering peritumor AT was then collected as the peritumor ADS. Peritumor ADS was diluted 1:10 in media with 0.1% FBS and applied to MDA-MB-436 cells for 24 h. Total cell lysates were analyzed by Western blotting. The ratio of phosho-S6 (S235/236)/total S6 trended towards a significant (*p* = 0.0525) increase in MDA-MB-436 cells treated with peritumor ADS from patients with BMIs ≥ 30 compared to peritumor ADS from patients with BMIs < 30. Western blot analysis of the remaining proteins showed their expression was not associated with BMI. JAG1 and ꞵ-actin were probed on separate blots with the same samples and ꞵ-actin was used to normalize samples. The BMIs associated with the specific patient IDs shown above the Western blot image are indicated in the Supplementary Materials (Supplementary Table S1). Results were analyzed by the student’s *t* test for statistically significant differences (*p* < 0.05). Data represent the mean signal ± SEM (error bars) (*n* = 5–6).

Next, we conducted experiments to determine the effect of BMI on peritumor breast ADS regulation of signaling in TNBC cells. To this end, MDA-MB-231 and MDA-MB-436 TNBC cells were treated with peritumor breast ADS for 24 h. We used antibodies that detect JAG1, phospho-ERK (Thr202/Tyr204), total ERK, phospho-NF-KB p65 (Ser536), total NF-KB, phospho-S6 (Ser235/236), and total S6 to probe total cellular extracts from TNBC cells for changes in JAG1, NF-KB, ERK, and mTOR complex 1 (mTORC1) signaling, respectively. The premise for assessing these pathways is based on prior studies showing that they regulate cancer [43,44,45,46]. Peritumor breast ADS from patients with BMIs ≥ 30 induced a statistically significant increase (by ~3-fold) in JAG1 relative to ADS from patients with BMIs < 30 in MDA-MB-231 cells (Figure 3). The phosphorylation of S6 (Ser235/236), ERK (Thr202/Tyr204), and NF-KB p65 (Ser536) was not changed in response to BMI (Figure 3). In contrast to MDA-MB-231, BMI was not associated with changes in the expression of JAG1 in MDA-MB-436 cells (Figure 4). In MDA-MB-436, the BMI effect on the regulation of phospho-S6 (Ser 235/236) in response to peritumor breast ADS was variable and trended toward being significantly (*p* = 0.0525) increased in response to BMI ≥ 30 relative to BMI < 30 (Figure 4). The phosphorylation of ERK (Thr202/Tyr204) in MDA-MB-436 cells was not significantly changed in response to BMI (Figure 4). The treatment of MDA-MB-436 cells with peritumor breast ADS from one patient with a BMI < 30 reduced the amount of phospho-ERK (Thr202/Tyr204) to levels that were below detection relative to the other samples (Figure 4). Given this outlier response, we did not include this sample in the phospho-ERK analysis. The phosphorylation of NF-KB (Ser536) was not changed in MDA-MB-436 in response to BMI (Figure 4). We assessed the effect of tumor grade on ADS regulation of signaling. The results show that tumor grade (grade 2 versus grade 3) was significantly associated with ADS-stimulated increases in JAG1 protein in MDA-MB-231, but not MDA-MB-436 cells (Supplementary Figures S5 and S6). The association of BMI with increased expression of JAG1 in MDA-MB-231 could in part explain why this TNBC cell line responded with increased invasive activity in response to peritumor ADS from patients with a high BMI, given that JAG1 can promote TNBC cell migration and invasiveness [40,47].

## 3. Discussion

Our study indicates obesity, as measured by BMI, is associated with increased TNBC cell migration and invasiveness in response to peritumor ADS (Figure 1). In line with an adipocyte effect, purified breast adipocytes stimulate TNBC cancer invasiveness [48,49,50]. However, ADS from differentiated breast adipocytes stimulate TNBC invasiveness independently of BMI [48]. The differentiation of breast stromal stem cells into adipocytes requires 4 days of cell culture with adipocyte induction media, which could obscure a BMI effect [48]. We utilized intact peritumor breast AT to assess secretome changes in the context of obesity, and the results indicate that obesity is associated with increased TNBC cell responsiveness to peritumor breast ADS, which is consistent with clinical studies showing obesity is associated with metastatic disease in breast cancer [20,51].

The cytokines and hormones secreted by AT are termed adipokines and they are the primary factors that mediate the obesity regulation of cancer [23,24,25]. Adipose-derived exosomes also promote cancer progression [52,53]. As a proof of principle, we measured the levels of four adipokines that are known to be secreted by AT [23]. Our finding that breast peritumor AT secretes leptin, IL-6, adiponectin, and FABP4 agree with prior publications [48,54]. We analyzed the levels of these adipokines based on BMI and identified an obesity effect on leptin and IL-6 but not adiponectin and FABP4 (Figure 2). Prior reports indicate that obesity does not affect the amount of leptin or IL-6 secreted from breast AT or purified breast adipocytes [48,54]. Adipokine secretion by breast AT from patients with breast cancer is highly variable, which is shown in our data (Figure 2) and by prior reports [48,54]. This variability could explain the inability to consistently show an obesity effect as defined by BMI on adipokine secretion by breast AT. The neoplastic changes on peritumor AT resulting from cancer are yet another factor that can cause adipokine variability. Adipocytes in direct contact with the tumor transition from normal adipocytes to cancer-associated adipocytes, which undergo delipidation and potentially dysregulation of adipokine secretion [31,55,56]. Given tumor heterogeneity, patients could have a unique intratumoral adipocyte function that leads to variable adipokine secretion. As our study was conducted with AT taken from peritumor tissue versus cancer-associated AT actively infiltrated by malignant cells, this could explain discrepancies between our study and prior reports. Baring complex biology, our finding that obesity is associated with increased leptin and IL-6 supports the observed effect of patient BMI on cancer cell invasiveness and migration, because these two adipokines stimulate breast cancer migration and invasiveness [41,42,57]. Many AT-secreted factors, such as exosomes and free fatty acids, have been linked to cancer progression [23]. We therefore hypothesize that leptin and IL-6 are not the only two factors that mediate the BMI effect on TNBC cell invasiveness. Future studies with additional patient samples are warranted to identify the potential cocktail of adipokines that mediate obesity regulation of TNBC cell invasion in patients.

Preclinical and clinical studies have linked JAG1 and its receptor NOTCH to increased TNBC progression [36,37,40,44,58]. JAG1 is produced in the tumor microenvironment and an increase in its levels promotes breast cancer cells to transition into a more aggressive and invasive phenotype [59,60]. The role of JAG1 in the tumor microenvironment agrees with the BMI effect we identified in our study that shows that a BMI increase is associated with increased expression of JAG1, migration, and invasiveness in MDA-MB-231 cells (Figure 1 and Figure 3). Proteins that increase JAG1 in the tumor microenvironment are proinflammatory cytokines, including IL-6 [61,62]. Leptin also increases the activity of the JAG1 receptor NOTCH1 in breast cancer cells [63,64]. Given these studies, it is possible that obesity-associated increases in IL-6 and leptin in peritumor breast ADS promote the expression of JAG1 in TNBC cells (Figure 2 and Figure 3). However, given the complexity of ADS, it is possible that other factors in ADS promote the expression of JAG1 in TNBC cells.

TNBC is a heterogeneous disease comprised of six subtypes of TNBC that cluster based on gene expression profiles [4]. The MDA-MB-231 and MDA-MB-436 cell lines belong to the mesenchymal-like morphology (MLM) subtype [4]. This subtype is enriched for stemness and invasiveness [4]. Our finding that MDA-MB-231 cells are responsive to peritumor AT and breast adipocyte ADS is consistent with prior studies [50,65]. To the best of our knowledge, the responsiveness of MDA-MB-436 cells to ADS has not been published. Our data show that the MDA-MB-436 cell line is less responsive to ADS than the MDA-MB-231 cell line (Figure 1). This difference is not due to subtype, because both cell lines cluster to the MLM subtype [4]. The BRAF, CDKN2A, KRAS, and TP53 genes are mutated in MDA-MB-231 cells, while the BRAC1 and RB1 genes are mutated in MDA-MB-436 cells [4]. The different mutational status might explain the different responsiveness to ADS. MDA-MB-231 cells are also more aggressive than MDA-MB-436 cells and MDA-MB-231 stem cells are functionally different than MDA-MB-436 stem cells, thus these molecular differences might contribute to MDA-MB-231 cells being more responsive to ADS than MDA-MB-436 cells [66]. The difference in TNBC cell responsiveness to ADS is consistent with complex relationship between obesity and TNBC in the clinical setting [67]. Moving forward it will be important to determine what receptors and pathways confer responsiveness of TNBC in the context of obesity. Such studies could identify molecular features in tumors that inform neoplastic behavior in obese patients.

Our results show an association between BMI, tumor grade, and the induction of JAG1 in TNBC cells (Figure 3, Supplementary Figure S5). Interestingly, all BMI ≥ 30 samples had grade 3 tumors, suggesting causation rather than coincidence, which can be investigated in a future study. Whether tumor grade alone could be a confounding factor in invasiveness, rather than BMI alone being the key factor is an important question; however, we did not have enough patients in this study to fully address the effect of grade. We did assay the effect of grade with the samples we had (Supplementary Figures S3–S6), and the data show no effect of grade that was independent of BMI for ADS in this study.

In summary, clinical studies show TNBC is more prevalent in obese than lean women [11,12,13]. Obese women are more prone to metastatic disease and the development of larger breast tumors than lean women [19,20,21,22]. These clinical findings are in line with the BMI effect of peritumor ADS-stimulated TNBC cell migration and invasiveness that we have identified in this study (Figure 1). Finding increased levels of leptin and IL-6 in ADS in association with BMI-defined obesity supports a direct BMI effect on peritumor AT (Figure 2). Given that JAG1 is linked to TNBC stemness and invasiveness and its expression in MDA-MB-231 cells is increased in response to ADS in association with BMI could reflect a new role for JAG1 in obesity. It is difficult to incorporate all the facets of human obesity in one study. Obesity induces endocrine, paracrine, metabolic, and immune alterations and also influences the efficacy of cancer drugs, and this complexity complicates the conclusions of this study that peritumor ADS plays a significant role in the behavior of TNBC cells in the context of obesity; however, the breast adipose tissue in this study is the most proximal breast adipose tissue that is not directly associated with cancer cells and thus the findings of this study do provide insight into paracrine regulation [23,68,69,70,71]. The limitations of this study that can be addressed in future studies include obtaining more clinical tissue so that the sample size is large enough to determine whether different grades of obesity play a role in paracrine regulation and conduct in vivo validation studies.

## 4. Materials and Methods

### 4.1. Cell Culture and Reagents

Triple-negative breast cancer cell lines (MDA-MB-231, MDA-MB-436) were purchased from American Type Culture Collection (ATCC) (Manassas, VA, USA). Cells were cultured in DMEM/F12 media supplemented with 10% fetal bovine serum (FBS) and penicillin streptomycin (P/S) antibiotics (P/S). Media, P/S, and FBS were purchased from Thermo Fisher Scientific (Waltham, MA, USA). The incubator was set at 37 °C with 5% CO_2_.

### 4.2. Breast Peritumor AT Derived Secretome

De-identified breast AT patient samples were obtained from women with breast cancer being treated at the Edwards Comprehensive Cancer Center at Cabell Huntington hospital in Huntington, West Virginia. The clinical characteristics of the patients with breast cancer are shown in Supplementary Table S1. The patients in this study were not homogenous in terms of clinical features. Patients were not pre-selected in this study, and samples were collected from consenting patients that were undergoing breast surgery as treatment for breast cancer. Breast adipose tissue samples were identified as non-cancer tissue located 5 to 15 cm away from the tumor, and paraffin sections were prepared and examined by a pathologist to determine that samples were cancer-free, as shown in representative paraffin sections in Supplementary Figure S1. The distance from the breast AT sample to the tumor ranged from 5 to 15 cm; therefore, the AT samples were not infiltrated by cancer cells and therefore not directly cancer associated but instead located near the tumor; therefore, the samples are referred to as being peritumor AT. Breast AT (~100 mg) was gently cut into five equal pieces using sharp surgical scissors, and clean AT pieces (5 pieces/5 mL) were cultured for 24 h at 37 °C/5% CO_2_. Media conditioned by peritumor AT was collected and briefly (~5 min) centrifuged to pellet tissue debris. Cutting, rinsing, and tissue culture were performed in serum-free cell culture media (RPMI/F12). Peritumor AT conditioned medium (termed the adipose-derived secretome (ADS)) was diluted 1:10 in DMEM/F12/0.1% fetal bovine serum and applied to TNBC cells. The 1:10 dilution dilutes potential harmful metabolites that might leach out from AT in cell culture. Patients with a BMI > 30 were categorized as obese. De-identified samples were obtained with patient consent.

### 4.3. Leptin and IL6 ELISA Assays

Leptin, IL-6, FABP4, and adiponectin concentrations were determined with ELISA Kits from Invitrogen (catalog numbers: KAC2281 & KHP0041) (Waltham, MA, USA) and R&D systems (Minneapolis, MN, USA) (catalog numbers: D6050B & DY3150-05), respectively.

### 4.4. Western Blot Analysis

TNBC cells (MDA-MB-231, MDA-MB-436) were plated in 60 mm dishes and allowed to reach confluency. Cells were treated with 10% ADS for 24 h. Cells were lysed in 400 µL 1X radio-immunoprecipitation assay (RIPA) buffer supplemented with protease and phosphatase inhibitors (Cell Signaling Technology, Danvers, MA, USA). Cells were incubated with RIPA buffer for 5 min and then transferred to microtubes. Cell lysates were sonicated for 15 s on ice. Cell lysates were boiled for 10 min in Laemmli buffer plus B-mercaptoethanol prior to SDS-PAGE. Proteins (12.5 µg) were transferred to polyvinylidene difluoride membranes (Bio-Rad Laboratories, Hercules, CA, USA). Membranes were incubated in primary antibody overnight (β-actin (Lot 20) 1:5000 dilution, NFKB 1:1000 dilution, phosphorylated NFKB (Ser536) (Lot 19) 1:1000 dilution, phosphorylated S6 (S235/236) dilution 1:1000, total S6 (Lot 13) dilution 1:1000, phosphorylated ERK (Thr202/Tyr204) (Lot 12) 1:1000 dilution, total ERK (Lot 28) 1:1000 dilution, and JAG1 (Lot 1) 1:500 dilution). Membranes were washed three times at five minutes per wash in 1X Tris-buffered saline 0.1% Tween 20 (TBST). Blots were incubated with secondary antibody (1:2000) for 2 h at room temperature while rocking and then rinsed three times with TBST (ten minutes per rinse). Proteins were detected using chemiluminescence (Bio-Rad Laboratories, Hercules, CA, USA). Bands were quantified using image lab software. Antibodies were purchased from cell signaling technologies (Danvers, MA, USA). For MDA-MB-231 cells, JAG1 and ꞵ-actin were probed sequentially on the same blot. For MDA-MB-436 cells, JAG1 and ꞵ-actin from the same samples were probed on separate blots because the ꞵ-actin signal was bleached out on the JAG1 blot, making quantification impossible. The expression of JAG1 was normalized to ꞵ-actin. Images of the original Western blots can be found in the Supplementary Materials (Supplementary Figure S6).

### 4.5. Migration Assay (Wound Healing Assay)

MDA-MB-231 and MDA-MB-436 cells were plated in 60 mm dishes and allowed to reach 100% confluency. Once the cells reached 100% confluency, they were washed with 1 mL of PBS. A 200 µL pipet tip was then used to create a cross-sectional wound and cells were treated with ADS. Images of cells within the center of the cross section were taken at time 0 and 24 h with a Leica Microscope. The area of the gap was calculated using the image J program. Fold change was calculated from the area of the gap measured at time 0 and time 24 h.

### 4.6. Invasion Assay

The Boyden chamber invasion kit from Millipore sigma was used (St. Louis, MO, USA) (CA #: ECM550). Fifty thousand cells were plated in the top chamber membrane in serum-free media. DMEM/10% FBS supplemented with 10% ADS was placed in the lower chamber as the chemoattractant. After 24 h, cells that migrated from the upper chamber to the lower chamber through the Matrigel-based membrane insert were rinsed and stained following the Boyden chamber protocol provided in the invasion kit.

### 4.7. Statistical Analysis

All data comparing BMI < 30 to BMI > 30 were analyzed by unpaired, two-tailed *t*-test. Raw *p*-values are shown in the figures, and *p* < 0.05 was considered statistically significant. Data comparing more than two groups were analyzed by ANOVA, and the data showed the groups were not statistically different. Statistics were performed using Graph-pad Prism (Version 10.3.1). Sample size was based on tissue availability; the data shown for migration and invasion represent 2–6 patients with multiple technical replicates, for ELISA, each patient represents a data point of 2–3 technical replicates, and each Western blot band represents a patient.

## Data Availability

Data are contained within the article and supplementary materials.

## Supplementary Materials

The following supporting information can be downloaded at: https://www.mdpi.com/article/10.3390/ijms251910592/s1.
