# Supplementary material for: High BMI Is Associated with Changes in Peritumor Breast Adipose Tissue That Increase the Invasive Activity of Triple-Negative Breast Cancer Cells
Source: Int J Mol Sci. 2024 Oct 1;25(19):10592. doi: 10.3390/ijms251910592 (PMC11476838; doi:10.3390/ijms251910592)
Supplement: Supplementary file 1 [file ijms-25-10592-s001.zip › ijms-3205683-supplementary.pdf]

Supplementary Materials: High (≥) Body Mass Index is Associated with Changes in Peritumor Breast Adipose Tissue that Increase the Invasive Activity of Triple-Negative Breast Cancer Cells

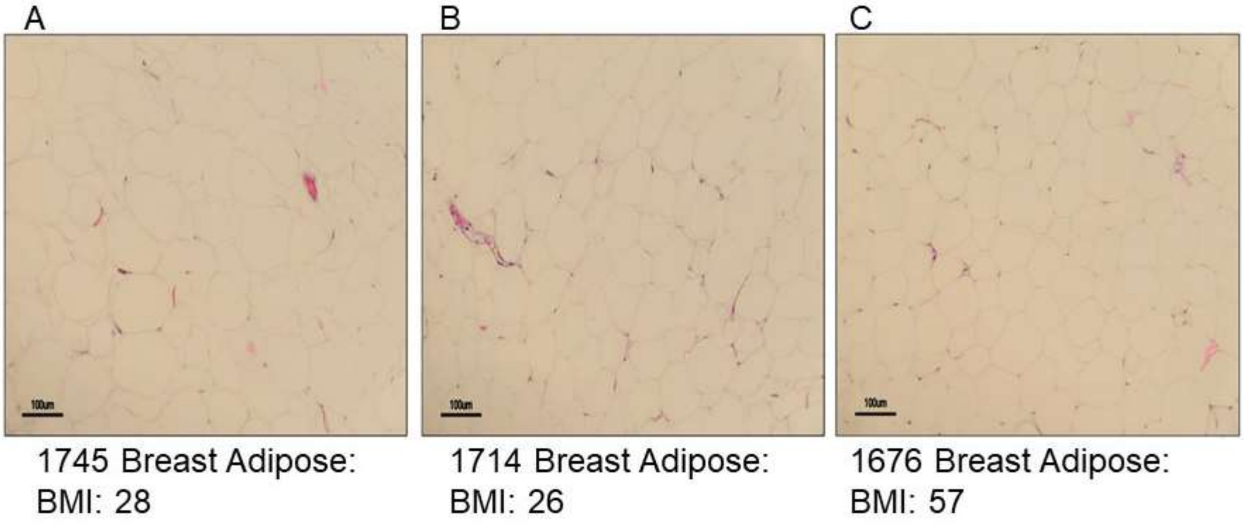

Supplementary Figure S1: Peritumor breast adipose tissue.

Hematoxylin and eosin (H&E) stains representative of peritumor adipose tissue that was used to generate peritumor breast adipose tissue derived secretome. These images are from three different patients with different BMIs (A) BMI of 28 (B) BMI of 26 and (C) BMI of 57.

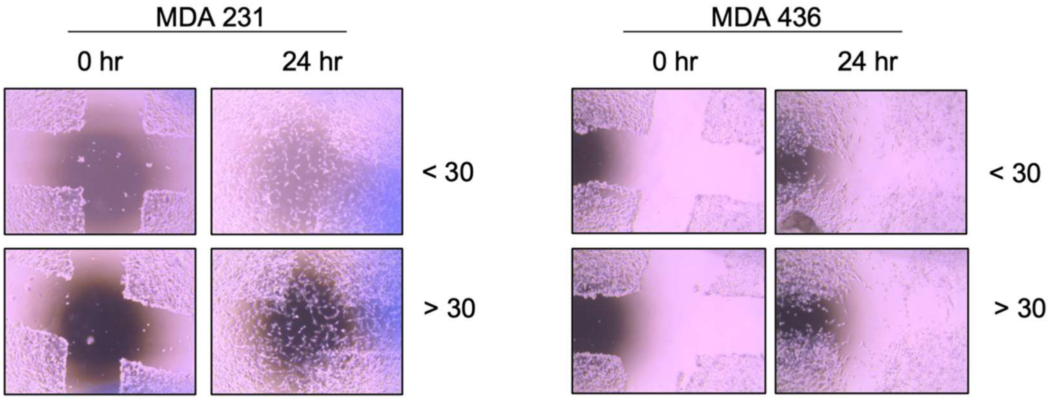

Supplementary Figure S2: Photographs showing cells from the migration data shown in Figure 1.

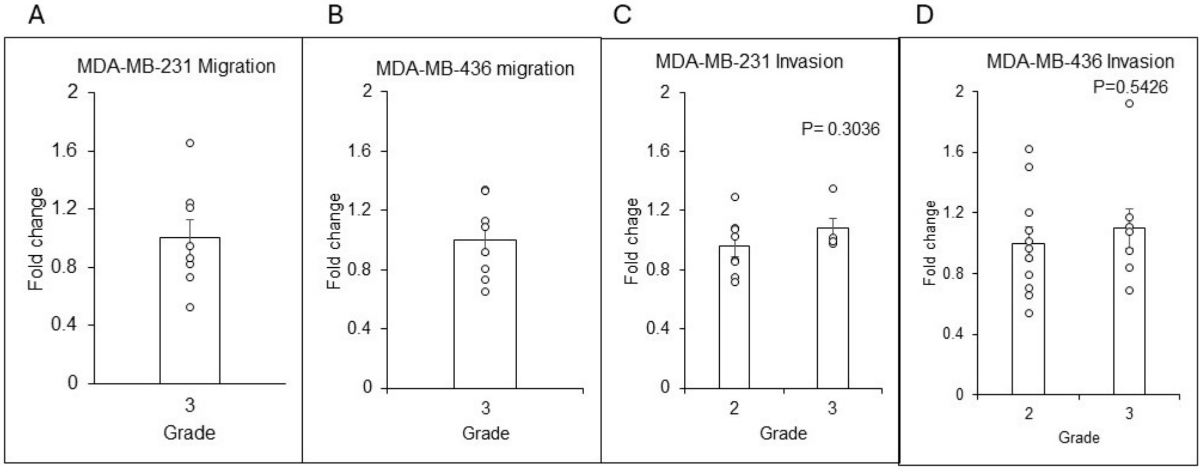

Supplementary Figure S3: Data from Figure 1 was used to assess the effect of tumor grade on peritumor AT-derived secretome on the migration of (A) MDA-MB-231; (B) MDA-MB-436; and invasiveness of (C) MDA-MB-231 and (D) MDA-MB-436. A & B. Tumor grade was not a factor in the cell migration studies, because samples were all Grade 3. **C & D.** Tumor grade (Grade 2 versus Grade 3) was not significantly associated with differences in ADS regulation of invasive activity. Results were analyzed by the student's t-test for statistically significant differences ($p < 0.05$). Data represents mean signal ± SEM (*error bars*) (n = 2 - 9).

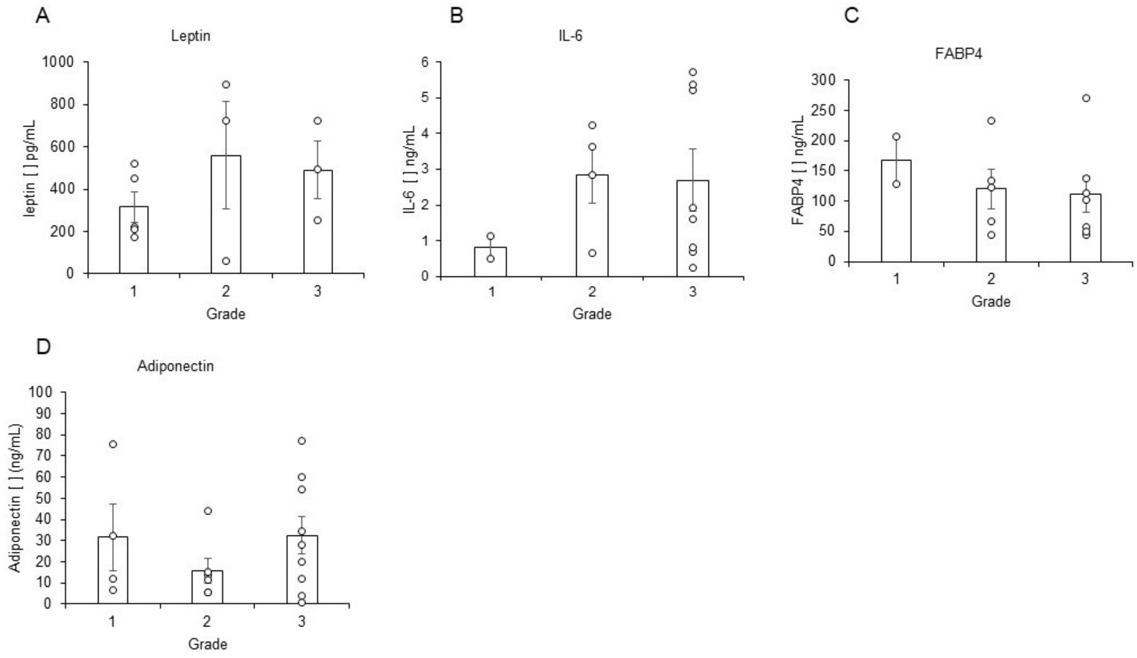

Supplementary Figure S4. Data from Figure 2 was used to assess the effect of tumor grade on the concentrations of leptin, interleukin-6 (IL-6), fatty acid binding protein 4 (FABP4), and adiponectin in peritumor AT-derived secretome (ADS). Tumor grade was not significantly associated with differences in the concentrations of (A) leptin, (B) IL-6, (C) FABP4 and (D) adiponectin in ADS. Data represents mean signal ± SEM (*error bars*) (n = 2 - 9).

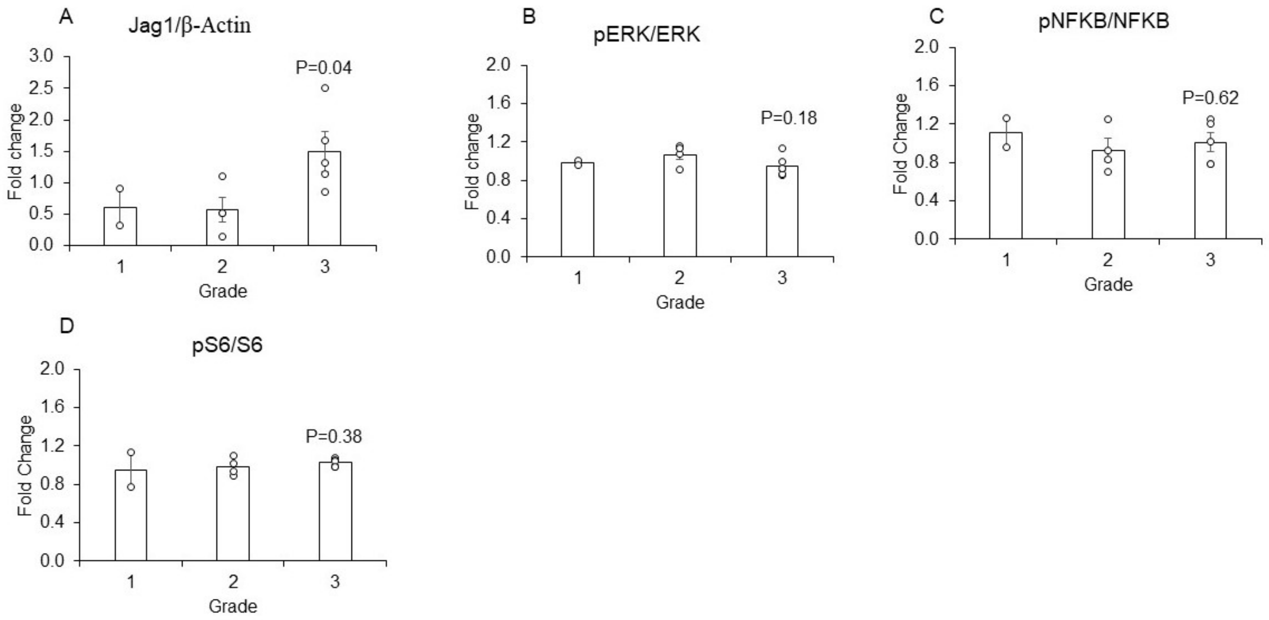

Supplementary Figure S5. Data from Figure 3 was used to assess the effect of tumor grade on peritumor ADS regulation of JAG1, phospho-ERK, phospho-NFKB and phospho-S6 in MDA-MB-231 cells. A. Tumor grade (Grade 2 versus Grade 3) was significantly associated with ADS-stimulated increases in JAG1 expression in MDA-MB-231 cells. Tumor grade (Grade 2 versus Grade 3) was not significantly associated with ADS regulation of **(B)** phospho-ERK (Thr202/Tyr204), **(C)** phospho-NF-κB (Ser235), and **(D)** phospho-S6 (S235/236). Results were analyzed by the student's t-test for statistically significant differences ($p < 0.05$). Data represents mean signal ± SEM (*error bars*) (n = 3 & 4).

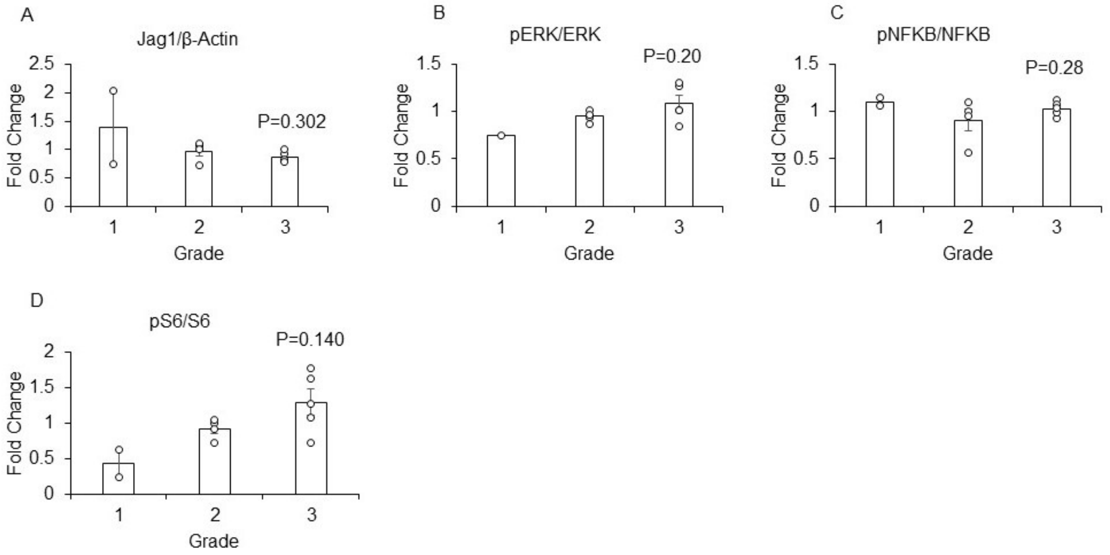

Supplementary Figure S6. Data from Figure 4 was used to assess the effect of tumor grade on peritumor ADS regulation of JAG1, phospho-ERK, phospho-NFKB and phospho-S6 in MDA-MB-436 cells. Tumor grade (Grade 2 versus Grade 3) was not significantly associated with ADS regulation of **(A)** JAG1, **(B)** phospho-ERK (Thr202/Tyr204), **(C)** phospho-NF-κB (Ser235), and **(D)** phospho-S6 (S235/236). Results were analyzed by the student's t-test for statistically significant differences ($p < 0.05$). Data represents mean signal ± SEM (*error bars*) (n = 3 & 4).

Supplementary Table S1. Clinical characteristics of patients in this study

Sample ID	BMI	Tumor grade	Age	Estrogen receptor (ER) expression
1059	22	3	52	+
1599	40	2	59	+
1252	20	2	68	+
1221	29	2	73	+
1613	31	3	70	+
772	23	2	48	+
1632	41	3	64	-
1292	23	Undetermined	34	+
902	24	1	44	+
1009	26	Undetermined	59	+
1697	26	1	42	+
1729	28	2	53	+
810	30	3	51	-
1683	36	2	45	+
928	32	3	59	+
1289	33	undetermined	52	+
1698	30	Undetermined	43	-
1676	57	3	39	-
657	22	1	44	+
545	22	1	60	+
897	29	2	54	+
368	24	1	80	+
650	23	3	58	+
788	33	1	60	+
1002	34	2	39	+
982	32	2	81	+
1700	33	3	28	+
1668	24	2	65	+
787	29	3	36	+
1710	32	3	60	+
1744	38	2	71	+
1716	28	3	60	+
871	37	3	64	+
